# Antitumor mechanisms of S-allyl mercaptocysteine for breast cancer therapy

**DOI:** 10.1186/1472-6882-14-270

**Published:** 2014-07-28

**Authors:** Hong Zhang, Kaiming Wang, Guimei Lin, Zhongxi Zhao

**Affiliations:** School of Pharmaceutical Sciences and Center for Pharmaceutical Research & DDS, Shandong University, Jinan, Shandong 250012 China

**Keywords:** S-allyl mercaptocysteine, Breast cancer, Apoptosis, Cell cycle arrest

## Abstract

**Background:**

S-allyl mercaptocysteine (SAMC), a water-soluble component derived from garlic, has been found to exert multi-antitumor activities. This study was to investigate the responsible molecular mechanisms of SAMC in human breast cancer cell lines.

**Methods:**

Sulforhodamine B assay was used to determine cell viability, flow cytometry was applied for the analysis of cell cycle and cell apoptosis, the change of protein was detected by Western blot.

**Results:**

It was found that SAMC exhibited an effective cell growth inhibition of human breast cancer cell lines MCF-7 (ER positive) and MDA-MB-231 (ER negative) in a dose- and time-dependent manner by inducing cell cycle arrested in G0/G1 phase, the block of cell cycle was associated with the up-regulation of p53 and p21. Furthermore, the SAMC-mediated cell cycle arrest was accompanied with promotion of apoptosis, as indicated by the changes in the nuclear morphology and expressions of apoptosis-related proteins. SAMC clearly triggered the mitochondrial apoptotic pathway as indicated by activation of Bax, decreased expression of Bcl-2 and Bcl-X_L_, and subsequent activation of caspase-9 and caspase-3.

**Conclusion:**

These results highlight the value of a continued investigation into the use of SAMC as a potential antitumor candidate for breast cancer.

## Background

Breast cancer is the top cancer in women both in the developed and developing world. The incidence of breast cancer has been increasing in the developing world and it has been the second leading cause of cancer death worldwide [1]. Each year there are more than 230,000 new cases and more than 30,000 women died from this disease [2]. Breast cancer is a heterogeneous disease with distinct clinical behaviors and molecular properties; in particular estrogen receptor (ER) positive and ER negative cancers are the two most distinct subtypes [3]. The treatment of breast cancer depends on various genetic, molecular and histological factors. In general, ER positive cells exhibit a higher proliferative capacity and distinct drug response than ER negative cells [4, 5]. However, ER negative cancer cells are generally more sensitive to chemotherapy, but associated with poor clinical outcomes [6, 7]. In clinic, the radiation therapy following breast-conserving surgery is recommended for early-stage breast cancers [8, 9]. Unfortunately, the majority of patients suffer from a high proportion of drug resistance and die of disseminated metastatic disease [8]. As a result, it becomes primary importance to search for more efficient and less toxic adjuvant therapeutic strategies that lead to better drug-free and overall survival.

The use of new therapeutic approaches based on plant-derived natural products for the prevention and treatment of cancer has gained a momentum in the past decades. Garlic (Allium sativum), a member of the lily family, is characterized by many sulfur-containing compounds, which make a main contribution to its bioactivities. A large amount of data indicates that garlic and its organosulfur compounds have anticarcinogenic activities. The use of garlic as anticancer dietary supplements had been reviewed by Fleischauer and Arab [10]. Individual organosulfur compounds in garlic have been studied in an attempt to identify the mechanisms of their anticarcinogenic activity especially for those oil-soluble compounds such as diallyl sulfide (DAS), diallyl disulfide (DADS), and diallyl trisulfide (DATS) [11–15]. For the water-soluble constituents such as S-allylcysteine (SAC) and S-allyl mercaptocysteine (SAMC), limited experimental studies also suggested that both water-soluble compounds can suppress cancer risk and alter the biological behaviors of various human tumors such as breast, prostate, bladder, colorectal and gastric cancers [16–21].

Li et al. examined the modulatory effect of SAC and SAMC on growth and glutathione cycle in two human cell lines MCF-7 and MCF-7(ras) [21]. It was found that SAC and SAMC produced an anti-proliferative response under both anchorage dependent and independent conditions as well as an alteration in glutathione level without significant concurrent changes in the glutathione metabolizing enzymes. Sigounas et al. has previously reported on the anti-breast cancer effects of SAMC and concluded that SAMC inhibited cell proliferation and reduced the viability of the breast cell line MCF-7 [19]. However, the mechanisms of inhibition effects in human breast cancer cell lines have not been clearly demonstrated. In this work, the antiproliferative effects of SAMC on both ER-positive (MCF-7) and ER-negative (MDA-MB-231) human breast cancer cell lines were investigated. The molecular mechanisms studied in this work include the assessment of the cell viability, cell migration, cell cycle distribution, and apoptosis which are mainly related to the expressions of tumor suppressor p53, p21, induced transcription of apoptosis-responsible genes such as Bax and Bcl-2 and activation of the caspase cascade. Our study revealed that SAMC inhibited cell proliferation by delaying the cell cycle at G0/G1 phase and triggered cell apoptosis through the mitochondrial (intrinsic) and death receptor (extrinsic) pathways.

## Methods

### Reagents

SAMC was synthesized and purified in our laboratory with a modified procedure as previously reported [22, 23]. A stock solution of SAMC (10 mM) was freshly prepared in PBS. Propidium iodide (PI), 4′,6-diamidino-2-phenylindole (DAPI), sulforhodamine B (SRB) were purchased from Sigma-Aldrich (St. Louis, Missouri, USA). Caspase-3/7, -8 and -9 activity, JC-1 and BCA protein assay kits were provided by Beyotime Institute of Biotechnology (Haimen, Jiangsu, China). Primary antibodies to p53, Bax, Bcl-2 and FADD (*fas*-associated protein with death domain) were obtained from Abcam (Cambridge, United Kingdom). The antibodies to cyclin D1, cyclin E1, cyclin A2, PCNA (Proliferating cell nuclear antigen), caspase-7, cytochrome c and Bcl-X_L_ were purchased from Epitomics, Inc. (Burlingame, CA). The antibodies to p21, E-cadherin and PARP (Poly (ADP-ribose) polymerase) were acquired from Merck Millipore (Darmstadt, Germany).

### Cell lines and cell culture

Human breast cancer cells MCF-7 and MDA-MB-231 were purchased from China Cell Bank (Shanghai, China). All cell lines were cultured in Dulbecco’s modified Eagle’s Medium with 10% fetal bovine serum, 100 U/mL of penicillin and 100 μg/mL of streptomycin and maintained in a humidified incubator of 5% CO_2_ at 37°C. When the growing cells reached approximately 70–90% confluence, they were treated with SAMC. The vehicle without SAMC was served as a control.

### Cell viability assay

The cytotoxicity of SAMC on human breast cancer cells MCF-7 and MDA-MB-231 was measured by SRB method [24]. The cells were seeded into 96-well plates for 24 h; then treated with SAMC for 24, 48 and 72 h. The treated cells were then fixed with 10% TCA for 1 h at 4°C, the 96-well plates were washed five times with distilled water and allowed to dry in the air. Each well was added with 100 μl of sulphorhodamine (SRB) solution and the staining was completed at room temperature for 15 min. The SRB stain solution was removed by washing the plates quickly with 1% (v/v) acetic acid five times, and the plates were dried in the air. The dried materials in each well were solubilized by adding 200 μl of 10 mM unbuffered Tris Base (pH 10.5). The cell viability was detected by measuring the absorbance at 540 nm on a plate reader (Safire2, TECAN, France). All experiments were repeated at least three times.

### DAPI staining

The human breast cancer cells MCF-7 and MDA-MB-231 were grown on 24-well plates for 24 h prior to the SAMC treatment and then treated with SAMC for 24 h. The treated cells were washed with PBS and fixed with cold methanol/acetone (1:1, store at -20°C) for 5 min at room temperature, the solution was removed and washed with PBS, and then incubated with the DAPI solution for 10 min at room temperature. Fluorescence images were captured using an Olympus model IX71 fluorescence microscope (Tokyo, Japan).

### Apoptosis analysis by annexin V and propidium iodide staining

The seeded human breast cancer cells MCF-7 and MDA-MB-231 in 6 well-plates were either treated with PBS or SAMC for 24 h. Detached and adherent cells were harvested and washed with PBS, then re-suspended in the binding buffer and stained with annexin V and propidium iodide (PI) according to the manufacturer’s instructions (Invitrogen, Carlsbad, USA). Apoptotic cells were analyzed by a Beckman Coulter model FC500 flow cytometer (Brea, CA, USA).

### Cell cycle analysis by flow cytometry

The human breast cancer cells MCF-7 and MDA-MB-231 were seeded in 6-well plates and grown overnight to achieve 80% confluence. After treatment with PBS or SAMC, all the cells were selected and washed with cold PBS, then fixed with iced 70% ethanol at 4°C overnight, centrifuged and washed with PBS. The washed cells were re-suspended and incubated with 0.5 mL of PBS containing 100 μg/mL RNase for 30 min at 37°C, and then incubated with 50 μg/mL PI for 30 min in the dark at 4°C. The cellular DNA content was analyzed by a Beckman Coulter model FC500 flow cytometer (Brea, CA, USA). Data were analyzed by using MODFIT and CELLQUEST software (Verity Software House, Topsham, Maine, USA).

### Wound closure assay

The breast cancer cells were seeded in 6-well plates and cultured until 90%-95% confluent. Three similar sized wounds were generated by scratching a gap using a sterile yellow pipette tip. Wounded monolayer cells were washed by PBS to clear cell debris and then incubated in a culture medium with or without SAMC. Images were captured under 40× magnifications every 8–12 hours using a phase-contrast microscope until the completed closure of the wound was observed in the vehicle-treated control.

### Assay for caspase-3/7, -8 and -9 activities

The assay for caspase-3/7, -8 and -9 activities was based on the ability of the active enzyme to cleave the chromophore from the enzyme substrates Ac-DEVD-pNA for caspase-3/7, Ac-LEHD-pNA for caspase-9, and Ac-IETD-pNA for caspase-8. Caspase activities were measured according to the manufacturer’s instructions. Levels of the released pNA were measured at 405 nm on a TECAN model Infinite M200 plate reader (Männedorf, Switzerland). All experiments were repeated at least three times.

### Analysis of mitochondrial membrane potential (ΔΨ_m_)

The mitochondrial membrane potentials (ΔΨ_m_) were analyzed by using a JC-1 assay kit according to the manufacturer’s instructions. Cells treated with carbonyl cyanide m-chlorophenylhydrazone (CCCP) were served as a positive control. Fluorescent intensity was measured by a Beckman Coulter model FC 500 flow cytometer (Brea, CA, USA).

### Western blot analysis

The whole-cell lysates were prepared by re-suspending cell pellets in the RIPA buffer. Equal amounts of proteins were loaded and separated by electrophoresis using SDS-PAGE (12%) and electro-transferred onto the polyvinylidene difluoride (PVDF) membrane. After blocking with 5% non-fat milk for 1 h at room temperature, the membranes were incubated with specific antibodies at 4°C overnight under slow migration. The antibodies to p53, p21, Bax, Bcl-2, Bcl-X_L_, FADD, PCNA, cyclin E1, cylcin D1, cyclin A2, caspase 7, cytochrome C, E-cadherin and PARP were used for corresponding protein development. Glyceraldehyde-3-phosphatedehydrogenase (GAPDH) was used as a housekeeping gene. Proteins of interest were visualized by an enhanced chemiluminescence detection system (Merck Millipore, Darmstadt, Germany) and the images were captured by Alphalmager HP system (Cell Biosciences, Inc., Santa Clara, CA, USA).

### Statistical analysis

Data from viability, cell cycle analysis and enzyme activity were obtained from experiments performed at least three times independently. Images were edited by Adobe Photoshop and figures were created by Origin 8.5. The student’s t-test was used to determine statistical differences between treated groups and controls, and P < 0.05 was considered statistically significant. The values were presented as mean ± SD. The significance level was calculated using one-way analysis of variance to assess the differences between experimental groups.

## Results

### Effects of SAMC on proliferation and cell cycle arrest of breast cancer cells

**Figure 1:**
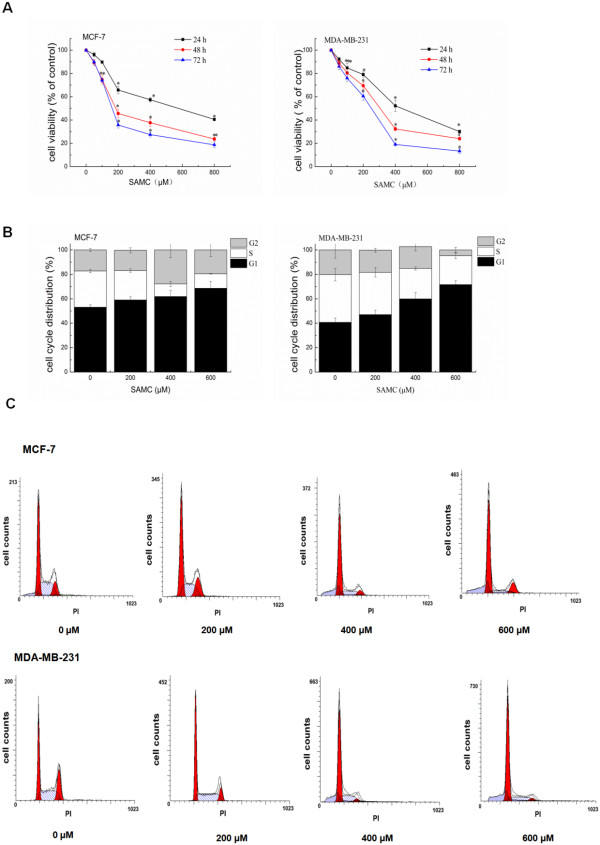
**The inhibitory effects and cell cycle progression of SAMC on human breast cancer cells.** The experiments were performed in triplicate and data are presented as mean ± S.D. of three independent experiments, *p < 0.05 when compared with control group. **(A)** Cytotoxic effects of SAMC on breast cancer cells for 24, 48 and 72 h measured by the SRB assay. **(B)** Cells were treated with SAMC (0–600 μM) for 24 h. For cell cycle analysis, cells were fixed, stained with PI and analyzed by flow cytometry. Quantitative analysis of percentage gated cells at G0/G1, S and G2/M phases were shown. All values were expressed as mean ± S.D. **(C)** Cell cycle analysis by flow cytometry, a hallmark of apoptosis was noted at high concentration of 400 and 600 μM.

The *in-vitro* anti-proliferation effects of SAMC on human breast cancer and were investigated on cancer cell lines ER-positive MCF-7 and ER-negative MBA-MD-231. As show in Figure 1A, SAMC significantly inhibited proliferation of breast cancer cells MCF-7 and MBA-MD-231 in a time- and dose- dependent manner. The IC_50_ value of SAMC was 148 μM for MCF-7 cells and 207 μM for MDA-MB-231 cells at 72 h.

The unrestrained cell proliferation leads to the generation of tumors, therefore, induction of cell cycle arrest has been appreciated as a target for the management of cancer [25, 26]. The DNA contents of MCF-7 and MDA-MB-231 cells after being treated with SAMC for 24 h were examined to confirm the proliferation inhibitory effects of SAMC on human breast cancer cells via the induction of cell cycle arrest. As show in Figure 1B, SAMC treatment induced a dose-dependent accumulation of cells in the G0/G1 phase and a corresponding decrease in S phase fraction in both breast cancer cell lines MCF-7 and MDA-MB-231. The accumulation of sub-G1 phase cells, a hallmark of apoptosis, was noted at high concentrations of 400 and 600 μM (Figure 1C). These results suggest that the proliferation inhibition of breast cancer cell lines MCF-7 and MDA-MB-231 by SAMC was through cell-cycle arrest in the G0/G1 phase.

**Figure 2:**
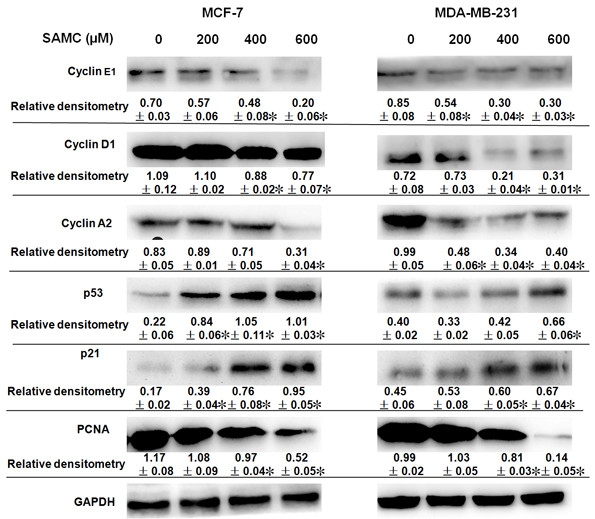
**The effects of SAMC on cell cycle by western blot analysis.** The cyclins, p53, p21 and PCNA were investigated with the GAPDH antibody served as a loading control. The experiments were performed in triplicate and all values were expressed as mean ± S.D., *p < 0.05 when compared with control group.

The intracellular localization of different cell cycle-regulating proteins also contributes to a correct cell cycle progression. Our Western blot assay results further demonstrate that SAMC decreased the expression of cyclin D1, cyclin E1 and cyclin A2, molecular makers of associated with the G1/S phase, in a dose-dependent manner in MCF-7 and MDA-MB-231 cells (Figure 2A). The p53 was the first tumor suppressor gene to be identified and believed to play an important role in regulating of cell cycle checkpoints [27]. The changes of p53 and its downstream target cyclin-dependent kinase inhibitor p21 were examined to determine their regulatory effects. As shown in Figure 2, induction of p53 was noticeable with increased concentrations of SAMC, and elevated p21 in SAMC-treated cells was correspondingly increased in a dose-dependent manner. Proliferating cell nuclear antigen (PCNA), a member of the so called DNA sliding clamp family, plays a coordinating role for numerous proteins involved in many processes involving DNA, such as DAN replication, DNA repair and cell cycle control [28–30]. The expression of PCNA was decreased following the treatment of MCF-7 and MDA-MB-231 cells with SAMC (Figure 2B). Thus, these results indicate that SAMC affected G0/G1 cell cycle checkpoints and caused a block of cell cycle progression.

### Effect of SAMC on breast cancer cell migration

**Figure 3:**
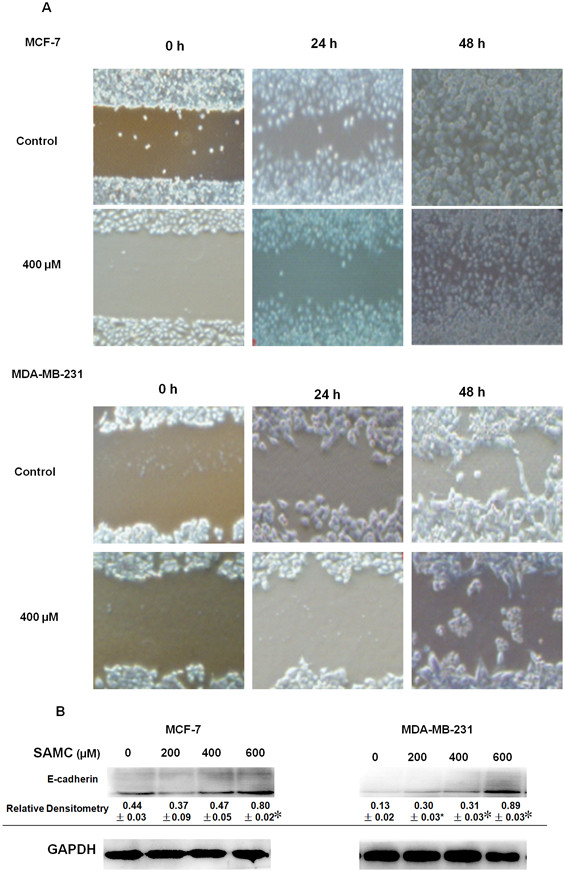
**The inhibitory effects of SAMC on cell migration. (A)** Differential cell migration rates were examined using a wound closure assay. **(B)** Western blot analysis of E-cadehrin with GAPDH antibody served as a loading control. The experiments were performed in triplicate and values are expressed as mean ± S.D., *p < 0.05 when compared with control group.

The metastatic stage was believed to be the main obstacle in the treatment of breast cancer, where breast cancer cell migration could be one of important characteristics during the process of cancer metastasis [31]. The migrations of human breast cancer cell lines MCF-7 and MDA-MB-231 after the treatment with SAMC were examined by using the wound closure assay. As shown in Figure 3A, the gap of wounds was gradually filled with migrating cells even almost completely closed (indicated by solid arrow) at 48 h after wound introduction, whereas the gap was still widely open (indicated by dotted arrow) in the controls. This inhibitory effect on cell migration was not the result of cell growth inhibition induced by these compounds as there was no significant difference in cell growth rate between the treated and control cells up to 48 hours post exposure time. Furthermore, considering the aberrant expression of E-cadherin is a common event in primary invasive ductal carcinomas that progress to develop distant metastases, we investigated the role of SAMC on regulating E-cadherin and found that SAMC was able to improving E-cadherin expression by western blot assay as shown in Figure 3B. These results indicate that SAMC treatment led to suppression of breast cancer cell migration, and may also be effective agents for the treatment of invasive cancers.

### SAMC induced apoptosis in breast cancer cells

**Figure 4:**
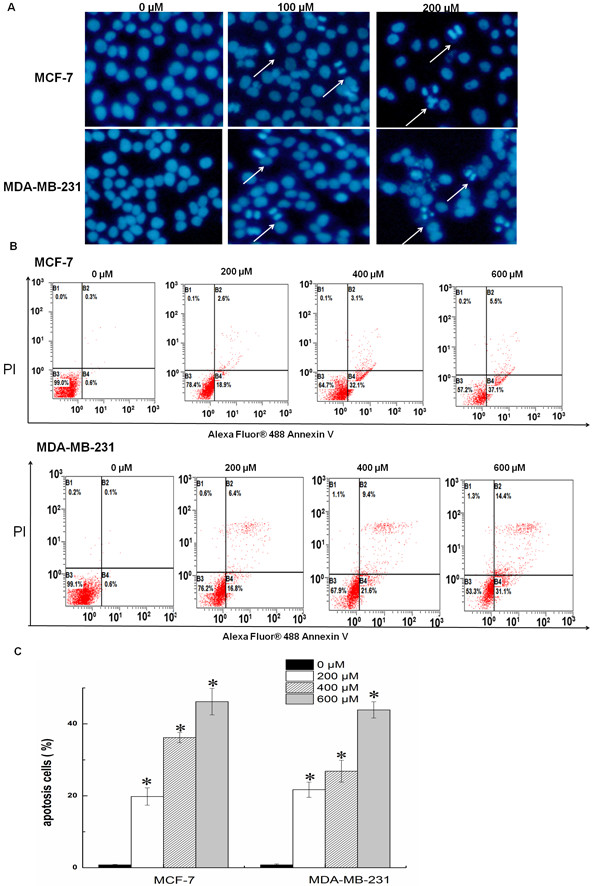
**SAMC induced apoptosis in breast cancer cells. (A)** Fluorescent micrographs of SAMC treated and untreated breast cancer cells after DAPI staining. **(B)** Flow cytometric analysis of SAMC induced apoptosis in MCF-7 and MDA-MB-231. **(C)** Statistical analysis of apoptosis. The experiments were performed in triplicate and values are expressed as mean ± S.D., *p < 0.05 when compared with control group.

DAPI staining was used to analyze the morphological changes of cells treated with SAMC. The condensed and fragmented chromatin characteristic of apoptotic cell death was observed as illustrated in Figure 4A. Quantification of the percentage of apoptosis induced by SAMC on breast cancer cells was performed by annexin V/PI staining and analyzed by a flow cytometer. As show in Figure 4B, SAMC treatment caused significant increases in the fraction of apoptotic cells in a dose-dependent manner, the percentage of apoptotic cells was increased from 1.1% to 45.5% in MCF-7 cells treated with 600 μM of SAMC (upper panel), and from 0.9% to 40% in MDA-MB-231 cells under same conditions (lower panel).

**Figure 5:**
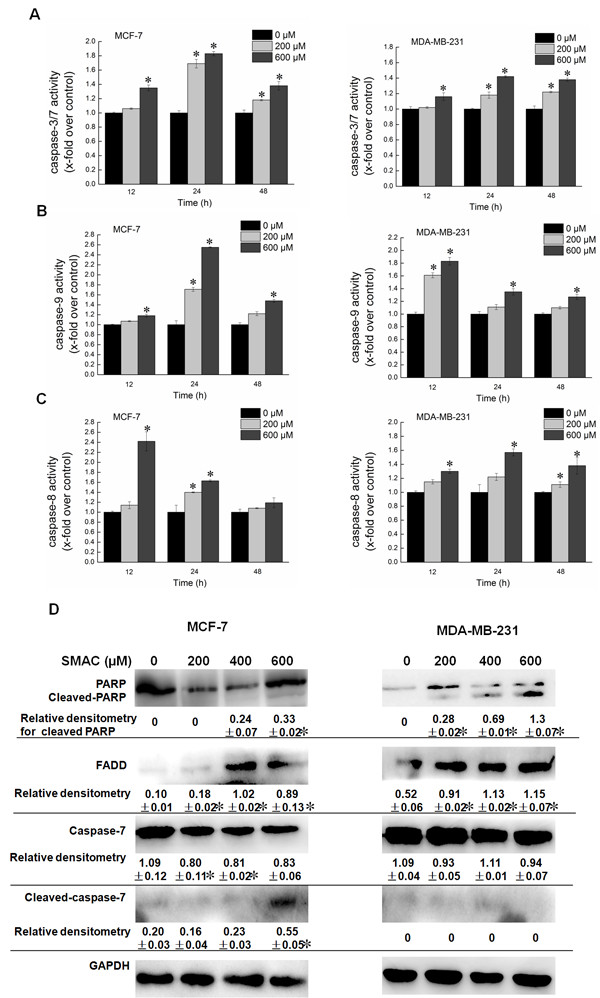
**Effects of SAMC on the activities of caspases, FADD and PARP.** Effects of SAMC on the activities of caspase-3/7, caspase-9 and caspase-8 in human breast cancer cell lines MCF-7 and MDA-MB-231 are shown in **(A)**, **(B)** and **(C)**, respectively. **(D)** The levels of caspase-7, PARP and FADD were measured by Western blot analysis. MCF-7 and MDA-MB-231 cells were exposed to various concentrations of SAMC. The values of optical density (OD) at 405 nm were determined in triplicate and all values are presented as mean ± S.D. of three independent experiments, *p < 0.05 vs. control cells.

Caspase activation represents the irreversible or execution stage of apoptosis [32, 33]. The involvement of caspases in apoptosis induction of SAMC was evaluated. The activities of caspase-3/7, caspase-9 and caspase-8 were also examined as shown in Figure 5A,B and C, respectively. It was found that caspase-3/7, caspase-9 and caspase-8 were all activated significantly when the breast cancer cell lines MCF-7 and MDA-MB-231 were treated with SAMC. These results indicate that both death receptor (extrinsic) and mitochondrial (intrinsic) pathways were involved in SAMC induced apoptosis. The Western blot analysis demonstrated that SAMC dramatically activated caspase-7 by increasing the cleaved caspase-7 level, which in turn led to the cleaved PARP in both MCF-7 and MDA-MB-231 cells. In addition, increased expression of FADD was also observed (Figure 5D); partially indicating that SAMC-triggered apoptosis was caspase-dependent.

### Mitochondrial dysfunction and regulation of expression of Bcl-2 family proteins caused by SAMC

**Figure 6:**
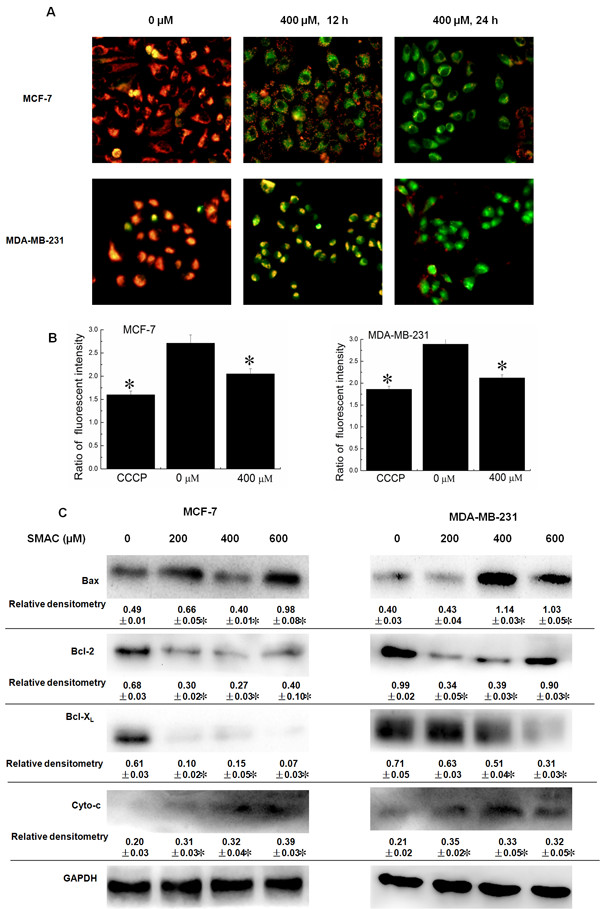
**Mitochondrial dysfunction and the effect on expression of associative proteins after SAMC treatment. (A)** The effect of SAMC on ΔΨ_m_ by JC-1 staining at the dose of 400 μM at 12 and 24 h. The photos were taken under 40× magnifications by a fluorescence microscope. The intensity of green fluorescence represents the loss of ΔΨ_m_. **(B)** The loss of ΔΨ_m_ analyzed by flow cytometry. **(C)** MCF-7 and MDA-MB-231 were treated with various concentrations of SAMC for 24 h, Bcl-2 family proteins such as Bcl-2, Bax, Bcl-X_L_ and cytochrome C were analyzed by the Western blot assay. The experiments were performed in triplicate and values are expressed as mean ± S.D., *p < 0.05 when compared with control group.

Mitochondrial membrane potentials regulate mitochondrial permeability, which plays an important role in triggering apoptotic pathways. The effect of SAMC on mitochondrial membrane potential ΔΨ_m_ was evaluated by JC-1 staining to determine whether mitochondrial dysfunction was involved in the apoptosis. As shown in Figure 6A, SAMC treated cells led to the dissipation of ΔΨ_m_ as indicated by increasing in green fluorescence emission. The flow cytometric analysis revealed that significant numbers of cells lose ΔΨ_m_ after the SAMC treatment (Figure 6B). Bcl-2 family proteins have been reported to regulate ΔΨ_m_. The expression of Bcl-2, Bax and Bcl-X_L_ were examined by the Western blot assay, the results reveal that SAMC treatment suppressed the expression of Bcl-2 and Bcl-X_L_, and increased the expression levels of Bax (Figure 6C). Further experiment was performed and cytosolic preparations were analyzed to examine whether the dysfunction of the ΔΨ_m_ resulted in the release of cytochrome *c*. The experimental results show that the amount of cytochrome c in the cytosol was significantly increased. These results suggest that the disruption of the mitochondrial membrane potential may be involved in SAMC-induced apoptosis.

## Discussion

Current conventional chemotherapy treatments are very expensive, toxic, and less effective in the majority cancer treatment. Plant-derived active components have been gaining more attention for their anticancer activities, over the last 25 years, approximately 63% of anticancer drugs introduced are natural products or can be traced back to a natural product source [34]. Garlic (Allium sativum), a member of the lily family, is widely cultivated and consumed worldwide. Many different health benefits have been ascribed to garlic for its diverse organosulfur compounds, and the anticarcinogenic actions of garlic have been reported by numerous epidemiological, clinical, and preclinical studies. At the same time, the use of garlic as the complementary and alternative medicine (CAM) by patients who are diagnosed with cancers is increasing. This phenomenon is without exception in the treatment of breast cancer.

In this study, we explored the molecular mechanisms by which SAMC induced cell apoptosis and cell death in breast cancer cell lines MCF-7 and MDA-MB-231. Our data demonstrate that SAMC exerted its inhibitory effects on cell proliferation of both ER positive and ER negative breast cancer cell lines MCF-7 and MDA-MB-231 by inducing G0/G1 cell cycle arrest, and simultaneously induced apoptosis in these two cell lines in a dose-and time-dependent manner.

It is well known that p53 plays a critical role in the induction of apoptosis, autophagy and cell cycle arrest. The CDKs and cyclin complexes were believed to influence the progression of cell cycle and its inactivation leads to cell cycle arrest; therefore, induction of cell cycle arrest has been appreciated as a target for the management of cancer [25–27]. This study revealed that SAMC enforced cell cycle arrest in the G0/G1 phase by activation of p53 and its key downstream target p21. Meanwhile, the expression levels of cyclin proteins such as cyclin D1 and cyclin E1 were down-regulated by SAMC. It is believed that p53 stimulated the transcription of different genes including p21, which is one of the cyclin-dependent kinase inhibitors. The induction of p21 resulted in CDK inhibition and cell cycle arrest, preventing the replication of damaged DNA [25]. It is likely that SAMC induced cell cycle arrest by p53 pathways as well as other signaling mechanisms since cell cycle checkpoints could be regulated by multi-factors. A variety of diseases including cancer can be caused by abnormalities in cell death control. Proteolytic enzymes such as caspases are important effective molecules in apoptosis. Activation of caspases in response to anticancer chemotherapy can be initiated through activation of the extrinsic (receptor) pathway or at the mitochondria by stimulating the intrinsic pathway [35]. The intrinsic pathway involves release of pro-apoptotic molecules from mitochondria to the cytosol such as cytochrome *c* that trigger the caspase cascade [36]. The main regulators of the intrinsic pathway are members of the Bcl-2 family proteins [37]. The extrinsic pathway relies on ligand activated recruitment of adaptor proteins by the death receptor and subsequent activation of caspase-8 [38].

**Figure 7:**
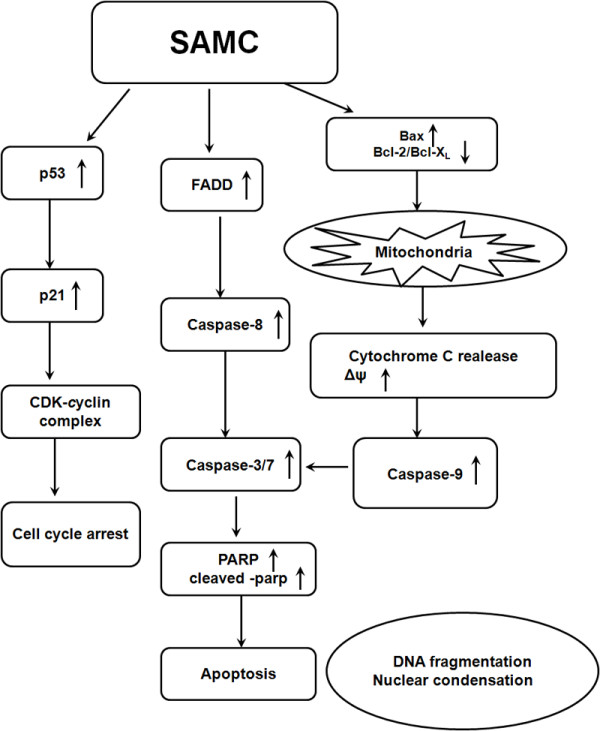
**The possible mechanisms of SAMC-induced cell cycle arrest and apoptosis in breast cancer cell lines MCF-7 and MDA-MB-231.**

Our investigation indicated that SAMC induced apoptosis of human cancer cell lines MCF-7 and MDA-MB-231 in a caspase-dependent way through extrinsic and intrinsic pathways (Figure 7). The mitochondrial function is regulated by Bcl-2 family proteins, which is thought to be key pathway for apoptosis. The mitochondrial dysfunction will lead to the reduction of mitochondrial membrane potential and generation of reactive oxygen species (ROS), which play an important role in cell apoptosis. Our results suggest that the Bcl-2 expression was decreased while the Bax expression was significantly increased, which was associated with the loss of ΔΨ_m_ and release of cytochrome *c*. In addition, the SAMC treatment of human breast cancer cell lines MCF-7 and MDA-MB-231 resulted in the activation of caspase-9 and caspas-3/7 as well as the increase of PARP, which lead to the intrinsic apoptosis. The extrinsic pathway of the apoptosis of human cancer cell lines MCF-7 and MDA-MB-231 after the SAMC treatment was revealed by the increase of FADD (*fas*) and the activation of caspase-8.

E-cadherin-mediated cell-cell adhesions limit cell motility and establish apical-basal polarity. Alterations of E-cadherin expression and disassembly of E-cadherin adhesion are consistently associated with the progression of carcinoma from a non-invasive to an invasive, metastatic phenotype [39]. In breast cancer, ER-positive tumors have been demonstrated to express normal amounts of the E-cadherin protein, and loss of ER and E-cadherin genes has been linked to disease progression of invasive breast carcinomas [40]. In this study, our results indicate that SAMC could inhibit the cell migration and restore or improve the expression of E-cadherin for both of ER-positive and ER-negative breast cancer cells, which could be a huge advantage in the chemoprevention and chemotherapy of breast cancer.

## Conclusion

This study elucidated the cellular mechanisms of SAMC as an anticancer agent for both ER-positive and ER-negative breast cancer cell lines MCF-7 and MDA-MB-231. Our results indicate that the inhibitory effect of SAMC against the breast cancer cell lines MCF-7 and MDA-MB-231 involved cell cycle arrest in the G0/G1 phase. Cell apoptosis was mediated by caspase activation and mitochondrial dysfunction. These findings support the continued investigation of SAMC as an alternative agent in the chemoprevention and chemotherapy for both ER-positive and ER-negative human breast cancer.
